# Optimization of Allelic Combinations Controlling Parameters of a Peach Quality Model

**DOI:** 10.3389/fpls.2016.01873

**Published:** 2016-12-20

**Authors:** Bénédicte Quilot-Turion, Michel Génard, Pierre Valsesia, Mohamed-Mahmoud Memmah

**Affiliations:** ^1^GAFL, INRAMontfavet, France; ^2^PSH, INRAAvignon, France

**Keywords:** model, optimization, genetic algorithm, QTL, ideotypes, *Prunus persica*, fruit

## Abstract

Process-based models are effective tools to predict the phenotype of an individual in different growing conditions. Combined with a quantitative trait locus (QTL) mapping approach, it is then possible to predict the behavior of individuals with any combinations of alleles. However the number of simulations to explore the realm of possibilities may become infinite. Therefore, the use of an efficient optimization algorithm to intelligently explore the search space becomes imperative. The optimization algorithm has to solve a multi-objective problem, since the phenotypes of interest are usually a complex of traits, to identify the individuals with best tradeoffs between those traits. In this study we proposed to unroll such a combined approach in the case of peach fruit quality described through three targeted traits, using a process-based model with seven parameters controlled by QTL. We compared a current approach based on the optimization of the values of the parameters with a more evolved way to proceed which consists in the direct optimization of the alleles controlling the parameters. The optimization algorithm has been adapted to deal with both continuous and combinatorial problems. We compared the spaces of parameters obtained with different tactics and the phenotype of the individuals resulting from random simulations and optimization in these spaces. The use of a genetic model enabled the restriction of the dimension of the parameter space toward more feasible combinations of parameter values, reproducing relationships between parameters as observed in a real progeny. The results of this study demonstrated the potential of such an approach to refine the solutions toward more realistic ideotypes. Perspectives of improvement are discussed.

## Introduction

Complex phenotypes of plants are not only regulated by both multiple interacting genes and environmental conditions but also by a series of interactions, competitions and feedbacks operating at different levels within the plant. In this context, modelers have the ambitions to synthesize information coming from complex genomics data sets, physiology and biochemistry with environment responses into mathematical models. Moreover, modeling can help to weave their way through this complexity and predict phenotypic consequences of changes at different levels (Hammer et al., 2006). The recently proposed concept of ‘crop systems biology’ that intends to bridge the gap between scales can help progressing in modeling crop genotype-phenotype relationships (Yin and Struik, 2010). The ultimate application goal of such integrated models is to better support design and breeding for complex traits in broad or specific environments. In other terms, the challenge is to optimize the strong genotype (G) × environment (E) interactions to design plant ideotypes that meet multiple conflicting breeding objectives (Martre et al., 2015).

To meet this challenge, several difficulties must be overcome. The first one consists in successfully integrating the genetic control in process-based plant models. Various research groups have worked on this integration for many years (see Yin and Struik, 2016 for examples), developing in particular a quantitative trait loci (QTL)-based modeling approach which aims to predict performance of any genotype in any environment. The principle is based on (i) the detection of QTL controlling values of the parameters of the process-based model and (ii) the back injection of the QTL-based parameter values into the process-based model. This approach enabled to simulate a simple phenotype of genotypes obtained via simulated sexual reproduction (Xu et al., 2012).

Next step concerns the identification of individuals displaying best performance in given conditions. Depending on the crop, performance may be described as a set of targeted traits that may prove to be antagonistic. In this case it is difficult to forecast which traits or processes to favor in order to get the best tradeoff. Using process-based models but lacking a dedicated method, one may run 1000s of simulations, combining values of parameters in all directions, without ever approaching optima. Fortunately, optimization algorithms are efficient tools to solve such problems. In a pioneering work, Hammer et al. (1996) proposed a general methodology for optimizing simultaneously the design and management of crop plants. In a case-study on optimizing maturity and density for a sunflower crop, they proved the potential power of the concept. Recent works making the connection of advanced optimization algorithms with process-based models confirmed that the approach is valuable to identify trade-off in complex systems (Qi et al., 2010; Quilot-Turion et al., 2012) and that specific adaptation of genotypes to local environmental conditions confers an advantage over broad adaptation (Hammer et al., 2014).

Nature-inspired optimization algorithms (e.g., genetic algorithms and particle swarm optimization algorithms) are increasingly used (deVoil et al., 2006; Letort et al., 2008; Qi et al., 2010; Ould-Sidi and Lescourret, 2011; Grechi et al., 2012; Kadrani et al., 2012; Quilot-Turion et al., 2012; Memmah et al., 2015). These algorithms both do not require any derivative information and enable exploring highly dimensional solution spaces. Amongst nature-inspired optimization algorithms, the Multi-Objective Evolutionary Algorithms (MOEAs) are the most known and effective. These optimization techniques are iterative, based on natural selection theory. They process a population of solutions in parallel and generate many feasible and non-dominated solutions, i.e., best tradeoffs between conflicting objectives (elements of the Pareto optimal set). Many MOEAs have been suggested over the last few decades. The most studied and the best performing variations among the MOEAs are the PESA (Corne et al., 2000), PESA-II (Corne et al., 2001), SPEA (Zitzler and Thiele, 1999), SPEA-II (Zitzler et al., 2001), NSGA (Srinivas and Deb, 1994), and NSGA-II (Deb et al., 2002) algorithms. The latter (Non-dominated Sorting Genetic Algorithm-II) is currently considered as the reference algorithm in the MOEAs community since it has proven to be one of the most efficient algorithms for solving multi-objective problems (Coello et al., 2007). The coupling of process-based models and MOEAs is progressively used to design model-based ideotypes. Examples of such approaches are: Letort et al. (2008) on beech trees, Qi et al. (2010) on maize, Lu et al. (2012) on wheat, Quilot-Turion et al. (2012) and Ould-Sidi et al. (2014) on peach, and Ding et al. (2016) on rice.

However, a major locking point persists on the extension of the second step to better consider the complex genetic architecture controlling the model parameters into account in the optimization scheme. Indeed, in a ‘Gene-to-Phenotype’ modeling approach, the simulations performed by Chenu et al. (2009) highlighted the importance of genetic architecture in the generation of real phenotypes. Taking into account the genetic constraints is the key to hope to actually create the solutions provided by the optimization procedure. Indeed the major limitation of the model assisted ideotype design is the lack of realism of these ideotypes that can never be obtained by a breeder, simply because they infringe physiological and genetic constraints.

One way is to include known genetic constraints in the definition of the space of parameter variation that is explored during the optimization step. This option is being explored by Picheny et al. (2016) using an indication of the domain of potential existence of the traits combinations. Another way to proceed is the direct optimization of allelic combinations included in a detailed genetic model taking into account linkage between close genes along the genome, pleiotropy of genes involved in the control of different parameters and epistasis between genes that have a specific effect when interacting. To our knowledge, this option has never been explored up to now. Only a first proof-of-concept study has been proposed by Letort et al. (2008) in this direction, with a reverse methodology of virtual QTL detection.

The present paper constitutes a first step toward the complete scheme of integration of process-based and genetics models associated with an optimization algorithm to design realistic ideotypes. A multi-objective optimization algorithm was used to find best allelic combinations to enhance three traits simulated by a peach fruit quality model. In a framework of a biparental progeny (two possible alleles at each locus), seven parameters of the process-based model were estimated for each of the 159 individuals and QTL detected for all of them. A genetic model including the allele effect of the wild parent at each locus for each parameter, pleiotropic effects of some loci and linkage between loci was developed. For sake of simplicity, epistasis was not considered in this study. The genetic model was combined with the ‘Virtual Fruit’ process-based model and coupled with the NSGA-II algorithm to solve the continuous (parameter real values) and the combinatorial (allelic binary values) optimization problems respectively. We compared the spaces of parameters obtained with different tactics and the phenotype of the individuals resulting from random simulations and optimization in these spaces. The features of the virtual phenotypes selected from the optimization steps were compared between the different tactics and discussed in sake of feasibility.

## Materials and Methods

### The Process-Based Model

The process-based simulation model used is the ‘Virtual Fruit’ model which integrates many sub-models dealing with fruit growth and quality elaboration (Génard et al., 2007). In this work we will focus on the sub-model describing the carbon (C) balance of a fruit-bearing stem. The description of the model is available in Supplementary Material (Model description S1 and **Supplementary Table S1**).

The leaf area is computed as the product of dry weight of the structural part of leaves and specific leaf area relative to the structural part of leaves (*SLA*, m^2^g^-1^). The latter is considered to be independent of tree growth and environment but under genetic control. The daily available pool of C assimilates consists of leaf assimilation plus possible C mobilized from reserves. The leaf photosynthesis rate may be affected by feedback inhibition through the leaf storage reserves. Fruit carbon assimilation and respiration are also considered. If required, reserves are mobilized first from the leafy shoot, then from the 1-year-old stem. Carbon is allocated according to organ demands and priority rules.

The incoming carbon flow into the fruit is partitioned into flesh, stone, and CO_2_ respiration (Quilot et al., 2005). The carbon is partitioned in the flesh into sugars and other fruit compounds globally considered (Quilot et al., 2004a).

The inputs of this model are global radiation, temperature, air relative humidity and stem water potential fixed to zero (no water limitation).

#### Dry Matter Growth

The potential fruit dry matter (*DM*) growth is modeled in terms of growing degree-days (GDD) using the following logistic equation:

$${DM}_{fruit}={DM}_{0}-\frac{A\cdot {DM}_{0}^{B}}{1+e^{{-RGR}_{ini}}\cdot \left(d_{j0}-P_{3}\right)}+\frac{A\cdot {DM}_{0}^{B}}{1+e^{{-RGR}_{ini}}\cdot \left(d_{j}-P_{3}\right)}$$

where:

*DM_fruit_*: is fruit dry mass (g) at date *d_j_*,

A and B: are parameters involved in the calculation of the logistic asymptote fixing final dry mass of fruit; their units are respectively (g) and (dimensionless),

RGR_ini_: is the initial relative fruit growth rate (GDD^-1^),

P_3_: is the date of the inflection point of the curve of fruit growth (GDD),

d_j_: is the date (GDD),

DM_0_: is the initial dry mass of fruit (g),

d_j0_: is the initial date (GDD) corresponding to DM_0_.

The values of these parameters are used to calculate the fruit carbon demand for each genotype.

The stone dry mass (*DM_stone_*) is computed as:

$${DM}_{stone}=W_{stone}\cdot \left(1-e^{\left(-k_{stone}\cdot {DM}_{fruit}\right)}\right)$$

where k_stone_ is a parameter (dimensionless) and W_stone_ (g) is a genotypic parameter which corresponds to potential maximal stone mass (plateau of the curve).

The stone ratio is then computed using (1) and (2).

#### Total Sugar Accumulation

The variation in total amount of carbon (g) in the fruit flesh as sugars (C_sugar_) is computed from the carbon used for dry flesh growth rate and the carbon used for synthesis of other carbohydrate compounds (e.g., starch, acids, structural carbohydrates, and proteins).

$$\frac{{dC}_{sugar}}{dt}=F_{C\,\sup p}\cdot \left(1-\frac{\alpha }{\alpha +\beta }\right)-k_{sugar}\cdot C_{sugar}$$

where F_Csupp_ is the flesh carbon supply (gC day^-1^) and k_sugar_ (day^-1^) is the relative rate of consumption of carbon as sugars in the fruit flesh for synthesis of compounds other than sugars. The parameters α (gC gDM^-1^) and β (gC gDM^-1^) are the respiration coefficient of fruit growth and the carbon concentration in fruit biomass, respectively.

The total sugar concentration, SU (g (100 g_FM_)^-1^), is computed as:

$$SU=100\cdot C_{sugar}/\left(\sigma_{TS}\cdot {FM}_{flesh}\right)$$

where σ_TS_ is an average carbon content of sugars (gC/g sugars) for sucrose, glucose, fructose, and sorbitol. FM_flesh_ is the flesh fresh mass (g).

The changes in flesh fresh mass are predicted as a function of turgor pressure by the Lockhart equation. The turgor results from the balance of water flows. The flow of water to the fruit is simulated considering full water availability for the plant. This flow is driven by differences of hydrostatic and osmotic pressures between stem and fruit and the fruit volume changes are predicted as a function of turgor pressure by the Lockhart equation. The flow of water leaving the fruit (transpiration) is calculated from skin conductance to water vapor.

The outputs used in this work are three fruit traits: dry mass of fruit (g), the part of stone in the fruit (in dry mass) and the total sugar concentration of the flesh. The model is driven by its parameters, which are constant over time and supposed independent of the environment. The simulations were performed for the year 2009 from 87 days after bloom (DAB) to 150 DAB. The parameter values were obtained from the literature or estimated for peach based on previous studies (Fishman and Génard, 1998; Lescourret et al., 1998; Génard et al., 2003; Lescourret and Génard, 2005).

We selected seven parameters: four involved in the *DM* growth, i.e., A,B,RGR_ini_,P_3_, two involved in the computation of the ratio between stone and fruit dry mass, i.e., k_stone,_W_stone_ and one involved in the calculation of carbon assimilation from sources, i.e., SLA (specific leaf area; m^2^ g^-1^). The choice of the first four parameters has been done based on deep sensitivity analysis of the used model. This choice was made based on two criteria: the main effect of each parameter on model outputs and the interaction terms with other parameters. We used different sensitivity methods and according to their results, we selected the parameters which show significant effects on the outputs of the model. The three remaining parameters were selected as being involved in some important biological processes.

### Simulating the Genotypic Variations of Fruit Quality in the Progeny

#### The Progeny

The peach genotypes studied come from a population obtained by two subsequent back crosses between *Prunus davidiana* P1908 (D) and *Prunus persica* cv. ‘Summergrand’ (S) and then cv. ‘Zéphyr’ (Z) as described by Quilot et al. (2004b). In the progeny, the proportions between the wild D allele and S alleles is expected to be 1/4 and 3/4 at one locus, one of the two Z alleles being always present at each locus.

#### Phenotyping

A total of 159 individuals of the progeny were monitored along fruit season in 2002 and/or 2005: fruit cheek diameter was measured once a week from the end of May to fruit maturity. At maturity, fruit were harvested. The flesh dry mass (*DM_flesh_*) was determined after drying flesh for 72 h at 70°C to constant weight. To compute dry fruit, flesh and stone masses for each monitored fruit, several allometric relationships between fruit diameter and fruit dry mass, stone dry mass and fruit dry mass, were used for each genotype (see Quilot et al., 2005 for details). Fruit flesh samples held at -80°C were then used for sugar measurement by HPLC following the procedure described in Gomez et al. (2002).

These experiments have enabled the acquisition of a dataset including kinetics of dry mass of fruit along fruit growth, of stone ratio in the fruit and of total sugar concentration of the flesh.

#### Parameter Estimations for the Progeny

The seven selected parameters of the model considered in this study were estimated for each individual of the progeny. SLA was estimated from the measurements of surface and mass of 20 leaves for each genotype. The six other parameters were estimated by fitting independently the three equations described in the ‘model section’ to the corresponding observed data (several fruit per genotype), using the ‘lnme’ R package which allows fitting non-linear mixed-effects models by maximum likelihood. This way we obtained a matrix including the values of the seven parameters for each of the 159 individuals of the progeny.

#### Simulations and Optimization

Using the process-based model described above and this matrix, we could simulate the kinetics of dry mass growth of fruit, stone ratio and total sugar concentration.

The model was used to find the set of the seven parameters allowing the optimization of the three traits of interest: maximize fruit dry matter mass (*DM*), minimize the stone ratio (*SR*), and maximize the sugar concentration (*SU*). This multiobjective optimization problem was formulated as follows:

{min⁡x(−DM(X),SR(X),−SU(X))Tsubject to X ∈ D

where X = (A,B,RGR_ini_,P_3_,k_stone_,W_stone_,SLA)^T^ is the vector of the parameters and D is the domain of variation of these parameters defined by lower and upper bounds. The negative sign before *DM* and *SU* objectives were introduced to ensure maximization of these objectives in a formulation based on minimization.

### The Genetic Model

#### QTL Analysis

The genetic map of the progeny monitoring the polymorphisms between the D and S genomes was built by Desnoues et al. (2016) and counts 340 independent loci. A subset of 151 individuals of the progeny which were both genotyped and phenotyped was used to perform the QTL analysis using R software (R Development Core Team, 2011, ‘Rqtl’ library).

#### The Genetic Model

For sake of proof of concept in this study, we kept all the QTL displaying a LOD score >1 (but only 1 per linkage group), so as to build a genetic model with a consequent number of loci. Thus a total of 37 QTL were kept (**Supplementary Figure S1**). All of the QTL detected for a parameter accounted for between 10 and 50% of the observed variation depending of the parameter (**Supplementary Table S2**). For each parameter, the effect of the presence of the D or S allele at each genetic locus linked to each QTL detected for the parameter was computed using a linear regression using ‘lm’ function in R. The resulting genetic model includes 31 different loci, since five loci are involved in two or three QTL of different parameters. This allows taking into account pleiotropic effect of some loci with contrasting effects on the different parameters. Those loci are positioned on the eight linkage groups and they are more or less distant from each other.

With any combination of alleles (0 or 1) at each of the 31 loci included in the genetic model, it is possible to calculate the corresponding values of the seven parameters of the process-based model. Thus, the genetic model was integrated in the ‘Virtual Fruit’ model. The values of the seven parameters are calculated from the alleles present at the QTL using the genetic model consisting of the following linear system of equations:

$$\left\{ \begin{array}{c} \mathrm{SLA=0.02103-0.00077}\times \mathrm{loc}[5]-0.00158\times \mathrm{loc}[\mathrm{10}]-0.00133\times \mathrm{loc}[\mathrm{15}]-0.00061\times \mathrm{loc}[\mathrm{21}]-0.00081\times \mathrm{loc}[26]\\ {\mathrm{RGR}}_{\mathrm{ini}}=\mathrm{0.00172+0.00059}\times \mathrm{loc}[1]+\mathrm{0.00038}\times \mathrm{loc}[\mathrm{14}]+\mathrm{0.00066}\times \mathrm{loc}[\mathrm{17}]+\mathrm{0.00119}\times \mathrm{loc}[\mathrm{22}]-\mathrm{0.00041}\times \mathrm{loc}[\mathrm{25}]+\mathrm{0.00079}\times \mathrm{loc}[\mathrm{31}]\\ A=8.29138+2.66318\times \mathrm{loc}[3]-4.17002\times \mathrm{loc}[7]+\mathrm{2.55744}\times \mathrm{loc}[\mathrm{12}]+\mathrm{2.51455}\times \mathrm{loc}[\mathrm{20}]+\mathrm{3.07437}\times \mathrm{loc}\left[\mathrm{23}\right]+\mathrm{3.55207}\times \mathrm{loc}\left[\mathrm{26}\right]\\ \mathrm{B=1.17567-0.25238}\times \mathrm{loc}[9]-0.20475\times \mathrm{loc}\left[27\right]\\ P_{3}=\mathrm{2695.74-227.64}\times \mathrm{loc}[\mathrm{14}]-\mathrm{168.95}\times \mathrm{loc}[\mathrm{18}]-260.25\times \mathrm{loc}[\mathrm{22}]+\mathrm{129}\times \mathrm{loc}[\mathrm{25}]-\mathrm{278.73}\times \mathrm{loc}\left[\mathrm{30}\right]\\ {\begin{array}{c} k \end{array}}_{\mathrm{stone}}=\mathrm{0.19725-0.03115}\times \mathrm{loc}[4]-\mathrm{0.02533}\times \mathrm{loc}[8]-\mathrm{0.0174}\times \mathrm{loc}[\mathrm{11}]-\mathrm{0.02218}\times \mathrm{loc}[\mathrm{13}]-\mathrm{0.02466}\times \mathrm{loc}[\mathrm{16}]-\mathrm{0.00724}\times \mathrm{loc}[\mathrm{28}]-\mathrm{0.01467}\times \mathrm{loc}\left[31\right]\\ {\begin{array}{c} W \end{array}}_{\mathrm{stone}}=\mathrm{3.86701+1.33592}\times \mathrm{loc}[2]+\mathrm{0.92848}\times \mathrm{loc}[6]+\mathrm{1.83601}\times \mathrm{loc}[\mathrm{14}]-\mathrm{0.22232}\times \mathrm{loc}[\mathrm{19}]-\mathrm{2.02367}\times \mathrm{loc}[\mathrm{24}]+\mathrm{1.33883}\times \mathrm{loc}\left[\mathrm{29}\right] \end{array}\right.$$

where the coefficients correspond to the additive effect of each QTL, and *loc*[x], with × from 1 to 31, take the value 0 or 1 depending on the presence of D or S allele at the QTL, respectively.

### Adding Constraints due to Loci Linkage

The probability to dissociate alleles of neighboring genes by obtaining genetic recombinants decreases gradually as the physical distance between the genes declines. The detailed analysis of the QTL results together with the genetic map dataset revealed the implication in the genetic model of loci closely linked. To take into account these supplementary constraints in the optimization step, we considered that distant loci less than 12.5 cM were inseparable. This led to the addition of 14 constraints and resulted in the definition of haplotypes including series of loci.

The enriched model (‘Virtual Fruit’ with genetic information) was used to find the best binary combinations of the 31 loci optimizing the three traits of interest: maximize fruit *DM* mass, minimize the stone ratio, and maximize the sugar concentration. This multiobjective optimization problem was formulated as follows:

$$\left\{ \begin{array}{c} {\begin{array}{c} \min_{\mathrm{loc}}\left(-\mathrm{DM}(\mathrm{loc}),\mathrm{SR}\left(\mathrm{loc}\right),-\mathrm{SU}\left(\mathrm{loc}\right)\right) \end{array}}^{T}\\ {\begin{array}{c} \mathrm{subject to loc}\in \left(\mathrm{0, 1}\right) \end{array}}^{31}\\ \forall i\in \{\mathrm{1,2,..., 14}\},\varepsilon -|\mathrm{loc}(\mathrm{insloc}\left(\mathrm{1,}i\right))-\mathrm{loc}(\mathrm{insloc}\left(2\mathrm{, i}\right))|>0 \end{array}\right.\,\left(5\right)$$

where *loc* is the vector of 31 loci, ε is a very small real, i.e., 1e^-9^ number and insloc is the set of 14 inseparable loci pairs given in the **Table 1**. The last formula represents the fact that for each of the 14 loci pairs, the two loci must take the same value (0 or 1) since they are considered inseparable and inherited from the same parent.

**Table 1:** Set of 14 inseparable loci pairs corresponding to loci distant of less than 12.5 cM.

Pair number	1	2	3	4	5	6	7	8	9	10	11	12	13	14
Involved locus numbers	4	6	7	10	13	14	16	17	18	21	23	24	25	28
	5	7	8	11	14	15	17	18	19	22	24	25	26	29


### The Optimization Procedures

We used NSGA-II algorithm to deal with both optimization problems formulated above. We used NSGA-II as it is considered in the optimization community as the reference thanks to its performances. Also, NSGA-II allowed us to tackle both continuous and combinatorial problems using the same algorithm while others MOEAs were developed especially for only one type. The NSGA-II algorithm works by randomly creating a parent population P0 and sorting it based on non-domination. To sort a population of N individuals according to the level of non-domination, each solution is compared with every other solution in the population. All individuals in the first non-dominated front are thus identified (F1). Then, the solutions of the first front are temporarily discounted, and the above procedure is performed again to find individuals for the next front (F2). The procedure is repeated for subsequent fronts (F3, F4, etc.) until all individuals are assigned to their ranks. The fitness is set to a level number: the lower the level the higher the fitness (F1 is the best). Next, the NSGA-II uses binary tournament selection, recombination, and mutation operators to create a child population Q0 of size N. At each subsequent generation, t, the algorithm merges the parent Pt and the child Qt populations into a combined population Rt of 2N individuals and sorts Rt according to non-domination, as described above. To create the next parent population, the NSGA-II uses the crowded comparison operator to select only N solutions from the Rt population. The new population Pt+1 is subsequently used for selection, crossover and mutation to create a new population Qt+1 of size N. The above procedure is continued until a predefined number of generations are created (Ould-Sidi et al., 2014).

The complexity of NSGA-II is O(MN^2^) where M is the number of objectives involved in the optimization problem to be solved and N is the size of the population. The interested readers can refer to the above cited references which give more details on NSGA-II. Parameters’ setting has been achieved based on the suggestions of the original version of NSGA-II for some parameters and on authors’ previous works for others. The NSGA-II parameters used in this paper are presented in **Table 2**.

**Table 2:** Parameters used in the NSGA-II algorithm for continuous and combinatorial variables.

Algorithm	Parameters	Values
Continuous variables	Population size	100
	Max generations	250
	Crossover probability	0.9
	Mutation probability	0.1
	Distribution parameter (for crossover)	20
	Distribution parameter (for mutation)	10

Combinatorial variables	Population size	100
	Max generations	250
	Crossover probability	0.9
	Mutation probability	0.01


### Description of the Stepwise Approach

We explored different spaces of parameters by random simulations and optimization following a stepwise approach. The space ‘parameters_progeny-fits’ corresponding to the parameter values observed in the progeny served as ‘observed’ reference in the study.

In a first step, we defined a parameter space (‘parameters_obs-bounds’) whose boundaries were fixed to the observed minimal and maximal values observed in the progeny. This parameter space was explored by random draw (500 sets of random values were drawn for the seven parameters and used to simulate the three targeted traits) and by an optimization procedure.

In a second step, a restricted parameter space (‘parameters_restricted’) was defined by fixing its boundaries to the minimal and maximal values made possible by the genetic model. Selecting all the alleles decreasing (increasing) the values of one parameter, we extracted the minimal (maximal) boundary of this parameter and defined a space representing the larger subset of values that one may hope rebuild from allele combinations. This space was also explored by random draw and optimization.

In a third step, on the basis of the genetic model we defined an allele space consisting in a matrix of 31 loci taking the values of either 0 or 1. This ‘alleles’ space was explored by random draw and optimization.

In the fourth and last step, linkage between loci was taken into account considering that loci closer than 12.5 cM could not be dissociated. This resulted in 14 constraints to include in the optimization procedure (‘alleles_optim_with-linkage’).

The number of simulations and solutions obtained by the optimization algorithm are presented in **Table 3** and the boundaries of the different spaces in **Table 4**. The phenotype of the individuals resulting from random simulations and optimization in these spaces were compared.

**Table 3:** Description of the eight datasets including parameter or allele values and the corresponding simulated phenotypes.

Model	Dataset	Exploration space	Boundaries	Number of data
Process-based model	parameters_progeny-fits			447 fruit from 159 genotypes
	parameters_random_obs-bounds	Parameter space	Minimal and maximal values from the progeny	500 distinct individuals
	parameters_random_restricted	Parameter space	Minimal and maximal values possible with the genetic model	500 distinct individuals
	parameters_optim_obs-bounds	Parameter space	Minimal and maximal values from the progeny	1193 distinct individuals
	parameters_optim_restricted	Parameter space	Minimal and maximal values possible with the genetic model	1370 distinct individuals
Integrated process-based and genetic models	alleles_random	Allele space	0 and 1 for all alleles	500 distinct individuals
	alleles_optim	Allele space	0 and 1 for all alleles	170 distinct individuals
	alleles_optim_with-linkage	Allele space	0 and 1 for sets of linked alleles (haplotypes)	14 distinct individuals


**Table 4:** Values of the boundaries of the parameter spaces and the allele space for the seven parameters.

Dataset	Boundary	*W_stone_*	*k_stone_*	*RGR_ini_*	*P_3_*	*A*	*B*	*SLA*

		**G**	**Dimensionless**	**GDD^-1^**	**GDD**	**g**	**Dimensionless**	**mg^-1^**
parameters_obs-bounds^∗^	Minimum	2.355	0.036	0.001	1203.528	0.771^∗^	0.01	0.013
	Maximum	12.885	0.378	0.008	2991.884	64.147	2.348	0.021

parameters_restricted	Minimum	1.62102	0.05448	0.00131	1760.17	4.12136	0.71854	0.01593
	Maximum	9.30625	0.19725	0.00533	2824.74	22.65299	1.17567	0.02103

alleles	Minimum	1.62102	0.05448	0.00131	1760.17	4.12136	0.71854	0.01593
	Maximum	9.30625	0.19725	0.00533	2824.74	22.65299	1.17567	0.02103


*^∗^In the parameters_optim_obs-bounds dataset, minimal value for parameter *A* was fixed to seven to avoid numerical problems.*

## Results

The quality fruit model was used in this study to simulate the three targeted traits: fruit dry mass, stone ratio and total sugar concentration.

### Simulating the Variability of a Progeny

**FIGURE 1:**
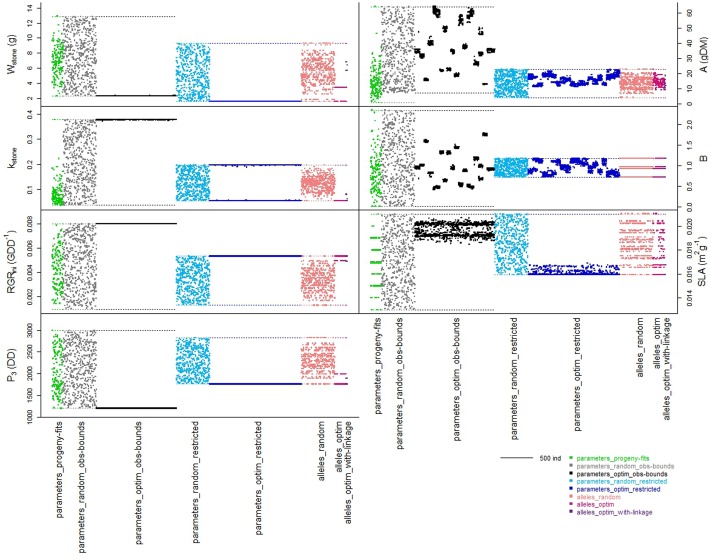
**Schematic representation of the distribution of the individuals from the different datasets within the spaces of the seven parameters of the process-based model.** Colors represent the different datasets organized along the *x*-axes. Width of each dataset depends on the number of individuals in the dataset. Colored dotted lines represent the boundaries (minimal and maximal parameter values) of the spaces respectively to each dataset.

On the basis of phenotype measurements, seven parameters of the process-based model were parameterized for the 159 individuals of the progeny. The parameter values and simulated traits were compiled in the ‘parameters_progeny-fits’ dataset. The distributions of the parameter values were sometimes uneven, especially for *k_stone_* and *A* (**Figure 1** _ green dataset). The set of the seven parameters enabled to simulate the large variability of phenotypes observed in the progeny for fruit dry mass, stone ratio, and total sugar concentration.

### Exploring the Parameter Space Whose Boundaries Are Defined by Extreme Observed Values in the Progeny

Within the ‘parameters_obs-bounds’ space, 500 sets of random values were drawn for the seven parameters and used to simulate the three targeted traits (**Figure 1** _ gray dataset).

**FIGURE 2:**
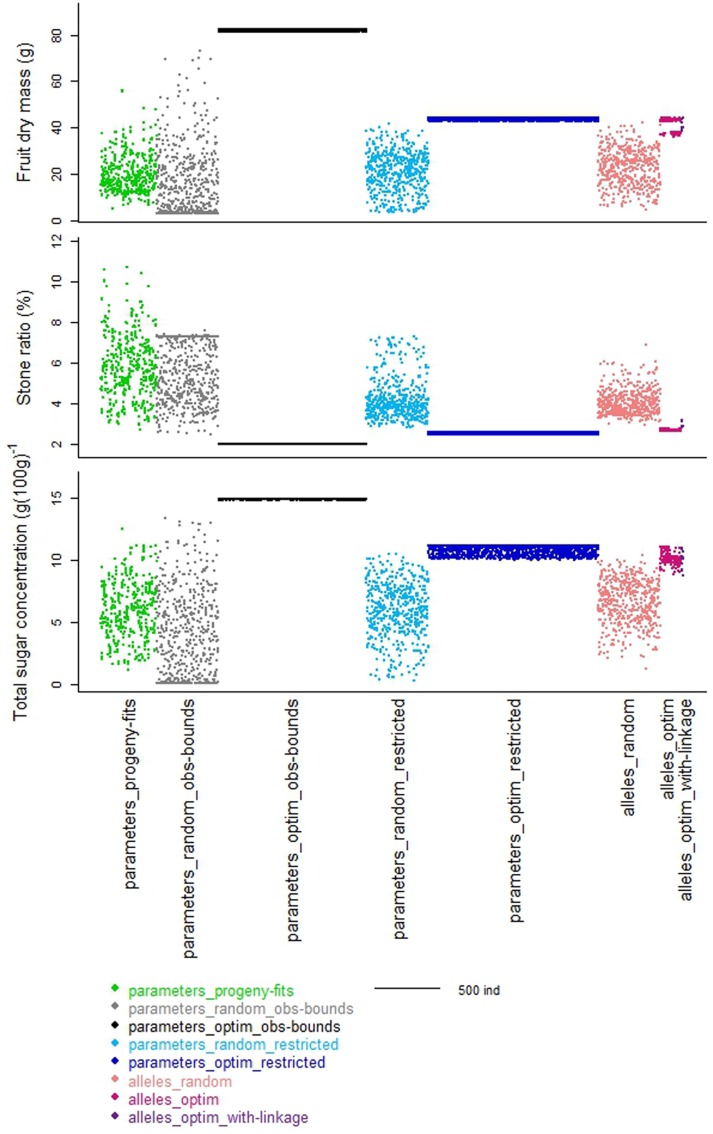
**Schematic representation of the distribution of the values of the three target traits taken by the individuals from the different datasets.** Colors represent the different datasets organized along the *x*-axes. Width of each dataset depends on the number of individuals in the dataset.

When looking to the stone ratio (**Figure 2**), part of the individuals from the ‘parameters_progeny-fits’ dataset was not reproduced by the ‘parameters_random_obs-bounds’ dataset. Hence, the individuals with relative large stone were not obtained by random drawing, suggesting that other parameters of the process-based model involved in stone elaboration may be genotype dependent.

The ‘parameters_obs-bounds’ parameter space was also explored thanks to the optimization procedure in order to find solutions which enhanced the three targeted traits (‘parameters_optim_obs-bounds’ dataset): 1193 distinct solutions were found. Four of the seven parameters had values nearly fixed toward one boundary of the parameter space, although the three other ones were distributed in a more or less large range of the space (**Figure 1** _ black dataset).

**FIGURE 3:**
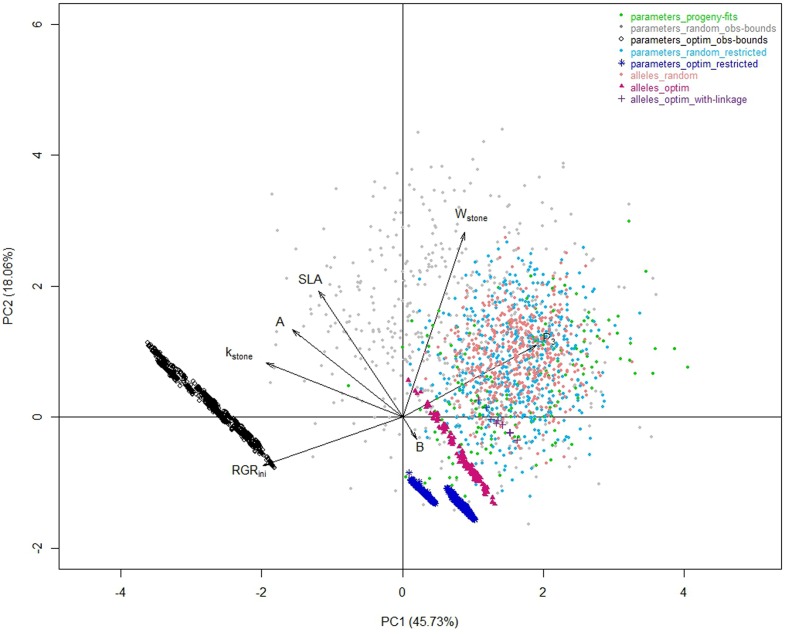
**Correlation plots of the seven model parameters for the first two principal components of a PCA analysis performed on the whole of the eight datasets**.

**FIGURE 4:**
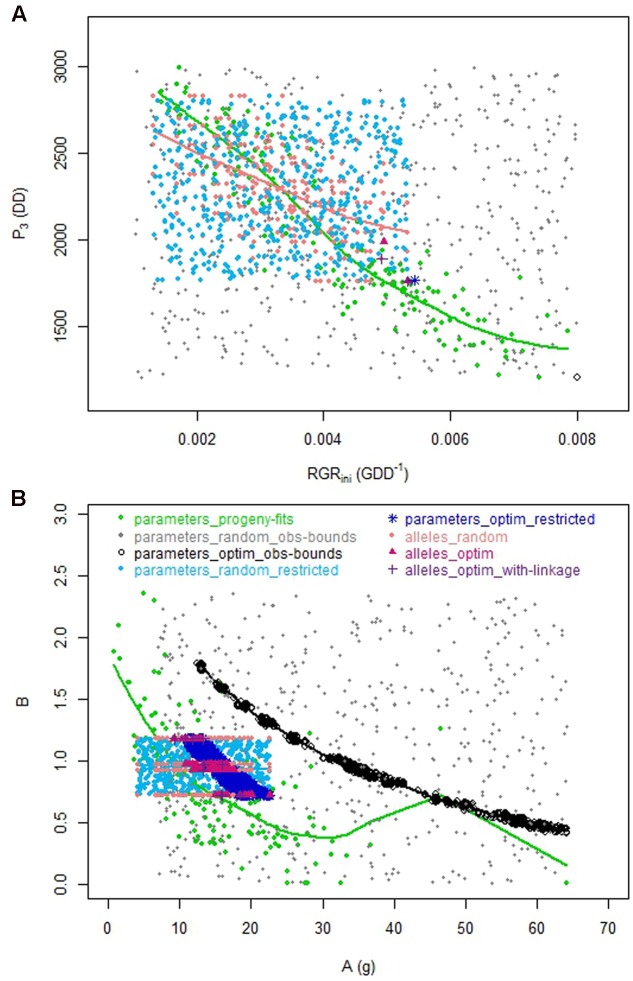
**Relationships between parameters for the eight datasets.** **(A)** Relationship between *P_3_* and *RGR_ini_*. **(B)** Relationship between *A* and *B*.

The optimization procedure allowed the detection of 1193 distinct individuals very similar in terms of phenotypes (**Figure 2**) but all better than the individuals obtained by random drawing of parameters. This emphasized the benefit of the optimization step to design combinations of parameters resulting in good phenotypes. It would require a very high density of random points to get individuals in this area. Indeed, best phenotypes stood in a zone hardly explored by random exploration suggesting highly refined combinations of parameters (**Figure 3**). The very large number of different parameter combinations compared to the very low variability of the resulting phenotypes pointed out the tight relationship between the parameters *A* and *B* (**Figure 4**), and the low influence of the parameter *SLA* on the targeted traits. In contrast the parameters *W_stone_*, *k_stone_*, *RGR_ini_*, and *P_3_* appeared as prevalent in the determination of the targeted traits.

**FIGURE 5:**
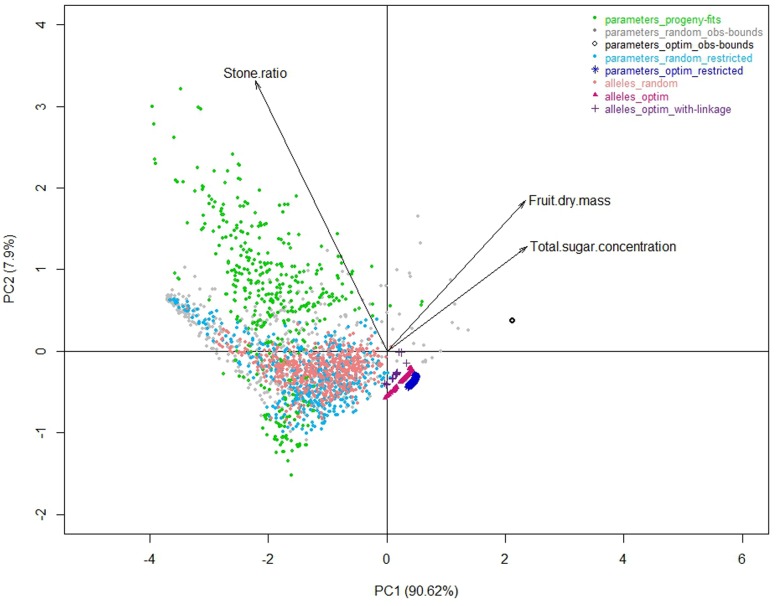
**Correlation plots of the three target traits for the first two principal components of a PCA analysis performed on the whole of the eight datasets**.

The best individuals of the random dataset and even more the optimized solutions were better than the observed individuals of the progeny. Indeed in this approach not one constraint was taken into account, neither physiological nor genetic, probably leading to unrealistic combinations of parameter values and outrageous phenotypes located in an unrealistic zone (**Figure 5** _ black empty circles compared to green points).

### Restraining the Parameter Space Thanks to the Genetic Model

The parameter values of the ‘parameters_progeny-fits’ dataset were used to detect QTL and define a genetic model including the wild D allele effect on each parameter, linkage information, and pleiotropic alleles. Because the QTL detected did not account for 100% of the observed variations of the parameters, the ‘parameters_restricted’ subset was generally included in the ‘parameters_obs-bounds’ space and smaller than it and thus it dramatically reduced the range of parameter combinations.

The random individuals from this parameter space were very similar to the individuals observed in the progeny for the three targeted traits (**Figure 2** _ light blue versus green datasets). The optimization procedure ended up again on a large number of very similar individuals. Comparing the 1370 optimized individuals obtained (**Figure 2** _ dark blue dataset) to the corresponding random landscape, similar conclusions to those stated previously on the strength of the optimization procedure can be drawn. Compared to the solutions obtained previously (‘parameters_optim_restricted’ versus ‘parameters_optim_obs-bounds’), the solutions were closer to the space explored by the “parameters_progeny-fits” dataset (**Figure 3**). Thus, the benefit procured by this step is the elimination of extreme individuals that may never be obtained.

### Considering Allelic Combinations Rather Than Parameters

The genetic model was combined to the process-based model in order to calculate the parameter values from allelic combinations. The ‘allele space’ consisted in a matrix of 31 loci taking the values of either 0 or 1. This way we switched from a continuous space of parameter values to a sparse space built from binary values of allele occurrence. In addition, the genetic constraints consisting in the pleiotropic effect of loci involved in the control of various parameters were respected.

Random drawing of allele combinations in this space allowed us to build the ‘alleles_random’ dataset. The more loci involved in the definition of a parameter, the more the resulting distribution of the values of this parameter was quantitative (**Figure 1** _ coral dataset). Hence the parameter *B*, controlled by only two loci in our genetic model may take four different values, whereas the parameter *k_stone_* controlled by seven loci displayed a quasi-continuous distribution. When regarding the distributions of the three traits, the random individuals were very diverse, almost as much as the ones from the ‘parameters_random_restricted’ dataset (**Figure 2** _ coral versus light blue datasets). Hence, the switch from a ‘parameter space’ to an ‘allele space’ did not reduce drastically the range of phenotypes.

The optimization procedure explored in record time this allele space of more than 2 billion solutions (2^31^). In contrast with the two previous datasets obtained from the optimization of the values of the parameters, the resulting distinct individuals were few, only 170 (‘alleles_optim’). The latitude of possible solutions was very narrow; however, those individuals outranged again the corresponding random landscape for the three targeted traits (**Figure 2** _ fuchsia versus coral datasets).

### Incorporating Linkage between Alleles

A step further in the approach was to include in the combined approach the information concerning the linkage between the loci controlling the parameters. As a first simple way, we considered that loci closer than 12.5 cM could not be dissociated, leading to a reduced number of possible combinations in the ‘allele space.’

The resulting solutions were very few, only 14 (‘alleles_optim_with-linkage’), and, as expected, they were worse than the ones from the ‘alleles_optim’ dataset, especially for the stone ratio (**Figure 2** _ purple versus fuchsia datasets). Compared to observed individuals from the ‘parameters_progeny-fits’ dataset (green), these optimized individuals reached another compromise of the three targeted traits: they displayed lower values than best observed values for dry fruit mass and total sugar concentration but part-cons lower values of stone ratio.

## Discussion

### Process-Based Models Generate Phenotypic Landscape

Using process-based models to generate the targeted traits is an excellent way to take into account physiological constraints between interacting processes and the influence of environmental factors on the expression of the phenotypes.

As an illustration, the peach fruit quality model transformed clouds of parameter values (**Figure 3**) into a very shaped landscape (**Figure 5**) which draws the space of possible solutions with a physiological point of view, constraining traits with compromises. Although, the individuals from the three random datasets looked nested with the ‘parameters_progeny-fits’ dataset in terms of values of parameters, they proved to be quite different when looking to the simulated traits resulting from the model. But they all aligned along borders that look impenetrable and which, without any doubt, mark physical, and/or physiological limits driving the fruit system.

In this study, we did not explore the impact of environmental factors on the realization of the phenotypes. Next interesting step would be to compare the parameters of best individuals obtained in contrasting growing conditions and thus reproduce and solve Genotype × Environment interactions.

### Adding Genetic Constraints to Improve Ideotype Realism

The genetic model built from QTL controlling the parameters of the process-based model enclosed different kind of information: allele effects, loci linkage, and pleiotropy. The combination of this genetic model with the ‘Virtual Fruit’ model enables the simulations of the phenotypes of virtual individuals with any combination of alleles at the loci controlling the parameters of the model, as done here in the ‘alleles_random’ dataset.

The genetic model allowed us to reproduce somehow the genetic links between the parameters that were observed in the progeny, which resulted in a reduction of the realm of possibility. Indeed, the relationship between *RGR_ini_* and *P_*3*_* observed within the ‘alleles_random’ dataset (coral dataset) reproduced quite well the tendency observed in the real progeny (green dataset), illustrating the strength of the genetic model (**Figure 4**). As for the relationship between *A* and *B*, the number of QTL detected for B did not allow reproducing properly the relationship. Hence, the switch from the ‘parameters_restricted’ space to the ‘allele space’ possibly added interesting effects on the reproduction of biological constraints conducting to the construction of more realistic phenotypes. Regarding data from the ‘parameters_optim_obs-bounds’ dataset (black dataset), they appeared out of the bounds of the observed relationships. In particular, due to the remoteness from the observed space for *RGR_ini_* and *P_*3*_*, and due to the shift of the relationship between *A* and *B*, the optimized individuals seemed unconceivable.

As previously mentioned, four parameters have been selected based on their main effects and interaction terms using sensitivity analysis. Three parameters have been selected as being involved in some important processes from agronomic and genetic points of view. Doing so, dependencies among parameters were expected. We actually identified correlations between *A* and *B* on one hand and *RGR_ini_* and *P_*3*_* on another hand. In addition, our results showed sometimes clear monotonic relationships between one trait to optimize and a parameter. In the absence of antagonist effect of the parameter on another objective, the optimization step rationally resulted in parameter values heading to the boundaries. However, we chose to keep working with the seven parameters (instead of proceeding to parameter reduction) as we were investigating the genetic architecture and effect of the QTL controlling these parameters. The QTL controlling dependent parameters were indeed not completely similar and reducing the model at this stage, as done by Ravi Kumar et al. (2009) could lead to disregard some potentially important QTL.

Taking into account the genetic model allowed restraining the space of solutions from an unrealistic landscape (gray dataset) to a more feasible one (coral dataset), and thus designing optimized solutions that are more likely to be obtained (purple versus black individuals).

Similar results were obtained by Chenu et al. (2009) when simulating the QTL impact on maize yield using a ‘Gene-to-Phenotype’ modeling approach. Indeed, considering pleiotropic effects of some QTL in their analyses resulted in substantial modifications of simulated yield and enhanced results compared to observed data. Subsequently, the authors underlined the fact genetic architecture is an important constraint that plays a key role in the generation of real genotypes. They largely discussed the importance of developing more complex genetic models to enhance the predictive capacity of the general approach. To improve the realism of these virtual genotypes, it is of course crucial that the genetic model is both complete and accurate, to better reproduce the continuing values of the parameters and enable rebuilding extreme values of the parameters.

One of the main difficulty to progress in this sense persists to be the estimation of the parameter values for 100s of individuals despite the elaboration of phenotyping platforms (Parent and Tardieu, 2014). Indeed, this is usually much more difficult than phenotyping macro-traits like fruit mass for example. However, this is needed to describe in a detailed manner the genetic architecture of the loci controlling the parameters, including complex effects such as pleiotropic and epistatic effects and even QTL × environment interactions.

### The Optimization Procedure to Identify Best Combinations

The optimization procedures used in this study proved to be very efficient to converge in the zones of the spaces explored that resulted in phenotypes outranging the corresponding random landscape, located at the margin of what could be impenetrable frontiers.

Finally, the solutions in terms of parameter values that appear most probable (‘alleles_optim_with-linkage’) fell into a space which was explored both by random individuals (‘alleles_random’) and real observed individuals. This is quite promising for the chance to be able to obtain them by crossing.

### Toward More Realistic Genetic Control of Parameters

The consideration of a bi-allelic progeny as the basis of the development of the genetic model is a simple case that dramatically reduces the genetic diversity taken into consideration. Especially in this case-study, only the effect of the presence or not of the wild allele was estimated. The case of an F2 population, for example, would at least add a little bit more of complexity in the genetic model, with three types of genotypes (two homozygotes and one heterozygote) and the issue of dominance versus additivity effects to consider. The latter issue was taken into account in Letort et al. (2008). An additional step will be to enlarge the diversity considered to develop an efficient breeding tool to select the best genitors for crossing.

### Unraveling the Difficulty to Obtain Desirable Combinations of Alleles

Finally, the study proposed here is imperfect since it did not take into account the probabilistic character of the linkage between loci. Thus, a further step to progress toward assisting plant breeding would be the development of an optimization procedure based on probabilistic rules that would allow building allele combinations according to distances between loci and thus rank the solutions according to the chance to obtain them. For this purpose and to develop a full breeding optimization system, two main ideas will be investigated in our future work. The first one will consist in integrating the stochastic (probabilities) constraints on loci linkage into the genetic model itself. Thus the values of the parameters predicted by the genetic model would take into account the linkage constraints. This is the option followed in quantitative genetics simulation systems as developed by Podlich and Cooper (1998) that can be applied in integrated approaches as done by Chapman et al. (2003) in a theoretical study for sorghum. Messina et al. (2011) applied this methodology within an operational breeding program in order to unravel best trajectories in breeding maize.

The second one will be to consider those constraints in the optimization step (mathematical formulation of the problem) and to deal with the new stochastic optimization problem under constraints of equality of alleles between near loci. The resulting problem will be harder to solve and will need specific tools (algorithms adapted for stochastic optimization problems) and techniques (constraints relaxation for example) to deal with.

## Conclusion

Developing an efficient tool to predict GxE environment and design ideotypes adapted to particular environmental conditions that the breeder has good chance to succeed to create is one important challenge for the coming years. The use of a process-based model combined with a genetic model and associated with an efficient optimization procedure is a promising path provided that the two models are robust and accurate.

## Author Contributions

BQ-T, MG, and M-MM conceived and designed the work; PV and M-MM devised the algorithms and performed the simulations; BQ-T analyzed the data; BQ-T, MG, and M-MM interpreted data; BQ-T, M-MM, and PV wrote the paper; MG revised it critically for important intellectual content; BQ-T, MG, PV, and M-MM approved the final version.

## Conflict of Interest Statement

The authors declare that the research was conducted in the absence of any commercial or financial relationships that could be construed as a potential conflict of interest.

## References

[B1] Chapman S. C. Cooper M. Poldich D. W. Hammer G. L. (2003). Evaluating plant breeding strategies by simulating gene action and dryland environment effects. *Agron. J.* 95 99–113. 10.2134/agronj2003.0099

[B2] Chenu K. Chapman S. Tardieu F. McLean G. Welcker C. Hammer G. (2009). Simulating the yield impacts of organ-level quantitative trait loci associated with drought response in maize: a “gene-to-phenotype” modeling approach. *Genetics* 183 1507–1523. 10.1534/genetics.109.105429 19786622 PMC2787435

[B3] Coello C. Veldhuizen D. V. Lamont G. (2007). *Evolutionary Algorithms for Solving Multi-Objective Problems.* New York, NY: Springer.

[B4] Corne D. W. Jerram N. R. Knowles J. D. Oates M. J. (2001). “PESA-II: region-based selection in evolutionary multiobjective optimization,” in *Proceedings of the Genetic and Evolutionary Computation Conference (GECCO-2001)*, San Francisco, CA.

[B5] Corne D. W. Knowles J. D. Oates M. J. (2000). “The pareto envelope-based selection algorithm for multiobjective optimization,” in *Proceedings of the Parallel Problem Solving from Nature PPSN VI: 6th International Conference*, eds Schoenauer M. Deb K. Rudolph G. Yao X. Lutton E. Merelo J. J. (Berlin: Springer), 839–848.

[B6] Deb K. Pratap A. Agarwal S. Meyarivan T. (2002). A fast and elitist multiobjective genetic algorithm: NSGA-II. *IEEE Trans. Evol. Comput.* 6 182–197. 10.1109/4235.996017

[B7] Desnoues E. Baldazzi V. Génard M. Mauroux J.-B. Lambert P. Confolent C. (2016). Dynamic QTLs for sugars and enzyme activities provide an overview of genetic control of sugar metabolism during peach fruit development. *J. Exp. Bot.* 67 3419–3431. 10.1093/jxb/erw169 27117339 PMC4892732

[B8] deVoil P. Rossing W. A. H. Hammer G. L. (2006). Exploring profit – sustainability trade-offs in cropping systems using evolutionary algorithms. *Environ. Model. Softw.* 21 1368–1374. 10.1016/j.envsoft.2005.04.016

[B9] Ding W. Xu L. Wei Y. Wu F. Zhu D. Zhang Y. (2016). Genetic algorithm based approach to optimize phenotypical traits of virtual rice. *J. Theor. Biol.* 403 59–67. 10.1016/j.jtbi.2016.05.006 27179460

[B10] Fishman S. Génard M. (1998). A biophysical model of fruit growth : simulation of seasonal and diurnal dynamics of mass. *Plant Cell Environ.* 21 739–752. 10.1046/j.1365-3040.1998.00322.x

[B11] Génard M. Bertin N. Borel C. Bussières P. Gautier H. Habib R. (2007). Towards a virtual fruit focusing on quality: modelling features and potential uses. *J. Exp. Bot.* 58 917–928. 10.1093/jxb/erl287 17283376

[B12] Génard M. Lescourret F. Gomez L. Habib R. (2003). Changes in fruit sugar concentrations in response to assimilate supply, metabolism and dilution: a modeling approach applied to peach fruit (*Prunus persica*). *Tree Physiol.* 23 373–385. 10.1093/treephys/23.6.373 12642239

[B13] Gomez L. Rubio E. Auge M. (2002). A new procedure for extraction and measurement of soluble sugars in ligneous plants. *J. Sci. Food Agric.* 82 360–369. 10.1002/jsfa.1046

[B14] Grechi I. Ould-Sidi M.-M. Hilgert N. Senoussi R. Sauphanor B. Lescourret F. (2012). Designing integrated management scenarios using simulation-based and multi-objective optimization: application to the peach tree–*Myzus persicae* aphid system. *Ecol. Model.* 246 47–59. 10.1016/j.ecolmodel.2012.07.023

[B15] Hammer G. L. Butler D. Muchow R. C. Meinke H. (1996). “Integrating physiological understanding and plant breeding via crop modelling and optimisation,” in *Plant Adaptation and Crop Improvement*, eds Cooper M. Hammer G. L. (Wallingford, CT: CAB International), 419–441.

[B16] Hammer G. L. Cooper M. Tardieu F. Welch S. M. Walsh B. Eeuwijk F. A. (2006). Models for navigating biological complexity in breeding improved crop plants. *Trends Plant Sci.* 11 587–593. 10.1016/j.tplants.2006.10.006 17092764

[B17] Hammer G. L. McLean G. Chapman S. Zheng B. Doherty A. Harrison M. T. (2014). Crop design for specific adaptation in variable dryland production environments. *Crop Pasture Sci.* 65 614–626.

[B18] Kadrani A. Sidi M.-M. O. Quilot-Turion B. Génard M. Lescourret F. (2012). *Particle Swarm Optimization to Design Ideotypes for Sustainable Fruit Production Systems.* Hershey, PA: IGI Global.

[B19] Lescourret F. Ben Mimoun M. Génard M. (1998). A simulation model of growth at the shoot-bearing fruit level I. Description and parameterization for peach. *Eur. J. Agron.* 9 173–188. 10.1016/S1161-0301(98)00035-5

[B20] Lescourret F. Génard M. (2005). A virtual peach fruit model simulating changes in fruit quality during the final stage of fruit growth. *Tree Physiol.* 25 1303–1315. 10.1093/treephys/25.10.1303 16076779

[B21] Letort V. Mahe P. Cournède P.-H. De Reffye P. Courtois B. (2008). Quantitaive genetics and functional-structural plant growth models: simulation of quantitative trait loci detection for model parameters and application to potential yield optimization. *Ann. Bot.* 101 1243–1254. 10.1093/aob/mcm197 17766844 PMC2710265

[B22] Lu C. Han J. L. Hu F. J. Qin T. G. (2012). Mathematical model of wheat stalk lodging-resistance during the later growth period. *Math. Pract. Theory* 42 46–53.

[B23] Martre P. Quilot-Turion B. Luquet D. Memmah M.-M. O.-S. Chenu K. Debaeke P. (2015). “Chapter 14 - Model-assisted phenotyping and ideotype design,” in *Crop Physiology*, 2nd Edn, ed. Calderini V. O. S. F. (San Diego, CA: Academic Press), 349–373.

[B24] Memmah M.-M. Lescourret F. Yao X. Lavigne C. (2015). Metaheuristics for agricultural land use optimization. A review. *Agron. Sustain. Dev.* 35 975–998. 10.1007/s13593-015-0303-4

[B25] Messina C. D. Podlich D. Dong Z. Samples M. Cooper M. (2011). Yield-trait performance landscapes: from theory to application in breeding maize for drought tolerance. *J. Exp. Bot.* 62 855–868. 10.1093/jxb/erq329 21041371

[B26] Ould-Sidi M.-M. Lescourret F. (2011). Model-based design of integrated production systems: a review. *Agron. Sustain. Dev.* 31 571–588. 10.1007/s13593-011-0002-8

[B27] Ould-Sidi M.-M. Quilot-Turion B. Kadrani A. Génard M. Lescourret F. (2014). The relationship between metaheuristics stopping criteria and performances: cases of NSGA-II and MOPSO-CD for sustainable peach fruit design. *Int. J. Appl. Metaheuristic Comput. (IJAMC)* 5 44–70. 10.4018/ijamc.2014070104

[B28] Parent B. Tardieu F. (2014). Can current crop models be used in the phenotyping era for predicting the genetic variability of yield of plants subjected to drought or high temperature? *J. Exp. Bot.* 65 6179–6189. 10.1093/jxb/eru223 24948682

[B29] Picheny V. Casadebaig P. Trépos R. Faivre R. Da Silva D. Vincourt P. (2016). Finding realistic and efficient plant phenotypes using numerical models. arXiv:1603.03238v1 [q-bio.QM].

[B30] Podlich D. Cooper M. (1998). QU-GENE: a simulation platform for quantitative analysis of genetic models. *Bioinformatics* 14 632–653. 10.1093/bioinformatics/14.7.632 9730929

[B31] Qi R. Ma Y. Hu B. de Reffye P. Cournede P.-H. (2010). Optimization of source-sink dynamics in plant growth for ideotype breeding: a case study on maize. *Comput. Electron. Agric.* 71 96–105. 10.1016/j.compag.2009.12.008

[B32] Quilot B. Génard M. Kervella J. Lescourret F. (2004a). Analysis of genotypic variation in fruit flesh total sugar content via an ecophysiological model applied to peach. *Theor. Appl. Genet.* 109 440–449. 10.1007/s00122-004-1651-7 15094993

[B33] Quilot B. Génard M. Lescourret F. Kervella J. (2005). Simulating genotypic variations of fruit quality in an advanced peach x *Prunus davidiana* cross. *J. Exp. Bot.* 56 3071–3081. 10.1093/jxb/eri304 16234284

[B34] Quilot B. Wu B. H. Kervella J. Génard M. Foulongne M. Moreau K. (2004b). QTL analysis of quality traits in an advanced backcross between *Prunus persica* cultivars and the wild relative species *P. davidiana*. *Theor. Appl. Genet.* 109 884–897. 10.1007/s00122-004-1703-z 15168024

[B35] Quilot-Turion B. Ould-Sidi M.-M. Kadrani A. Hilgert N. Genard M. Lescourret F. (2012). Optimization of parameters of the ‘Virtual Fruit’ model to design peach genotype for sustainable production systems. *Eur. J. Agron.* 42 34–48. 10.1016/j.eja.2011.11.008

[B36] R Development Core Team (2011). *R: A Language and Environment for Statistical Computing*. Vienna: The R Foundation for Statistical Computing Available at: http://www.R-project.org/

[B37] Ravi Kumar S. Hammer G. L. Broad I. Harland P. McLean G. (2009). Modelling environmental effects on phenology and canopy development of diverse sorghum genotypes. *Field Crops Res.* 111 157–165. 10.1016/j.fcr.2008.11.010

[B38] Srinivas N. Deb K. (1994). Muiltiobjective optimization using nondominated sorting in genetic algorithms. *Evol. Comput.* 2 221–248. 10.1162/evco.1994.2.3.221

[B39] Xu L. Ding W. Henke M. Kurth W. Zhu J. Buck-Sorlin G. (2012). “Simulating superior genotypes for plant height based on QTLs: towards virtual breeding of rice,” in *Proceedings of the IEEE Fourth International Symposium on Plant Growth Modeling, Simulation, Visualization and Applications (PMA)*, Shanghai, 447–454.

[B40] Yin X. Struik P. C. (2010). Modelling the crop: from system dynamics to systems biology. *J. Exp. Bot.* 61 2171–2183. 10.1093/jxb/erp375 20051352

[B41] Yin X. Struik P. C. (2016). *Crop Systems Biology Narrowing the Gaps Between Crop Modelling and Genetics.* New York, NY: Springer.

[B42] Zitzler E. Laumanns M. Thiele L. (2001). “SPEA2: improving the strength pareto evolutionary algorithm for multiobjective optimization,” in *Evolutionary Methods for Design Optimization and Control with Applications to Industrial Problems*, eds Giannakoglou K. C. Tsahalis D. T. Papailiou K. D. Fogarty T. (Barcelona: International Center for Numerical Methods in Engineering), 95–100.

[B43] Zitzler E. Thiele L. (1999). Multiobjective evolutionary algorithms: a comparative case study and the strength Pareto approach. *IEEE Trans. Evol. Comput.* 3 257–271. 10.1109/4235.797969

