# Supplementary material for: Optimization of Allelic Combinations Controlling Parameters of a Peach Quality Model
Source: Front Plant Sci. 2016 Dec 20;7:1873. doi: 10.3389/fpls.2016.01873 (PMC5167719; doi:10.3389/fpls.2016.01873)
Supplement: Supplementary file 1 [file Data_Sheet_1.docx]

Supplementary Material

**Optimization of allelic combinations controlling parameters of a peach quality model**

**Quilot-Turion B.^*^, Génard M., Valsesia P., Memmah M-M.**

*** Correspondence:** Quilot-Turion Bénédicte: [benedicot-turion@inra.fr](mailto:benedicte.quilot@avignon.inra.fr)

**Model description S1.** Main equations used in the model to predict fruit growth from C assimilation and allocation. Model parameters are defined in Table S1.

**Leaf assimilation**

The total leaf area LA (m²) of a stem is computed from the dry mass of the structural part of leaves, WS_l_ (g), and the specific leaf area, *SLA* (m^2^ g-^1^), as follows:

LA = WS_l_ x *SLA* (1)

Light-saturated leaf photosynthesis P_l_^max^ (μmol CO_2_ m^-2^ s^-1^) is modulated by the level of reserves in the leaves (R). ***p_1_*** (μmol CO_2_ m^-2^ s^-1^) is the maximum of light-saturated leaf photosynthesis in the absence of leaf reserves:

(2)

Photosynthesis per unit leaf area and per unit time is calculated from the photosynthetically active photon flux density (PPFD, μmol photon m^-2^ s^-1^):

P_l_ = [ ( P_l_^max^ + *p3*) x ( 1 – e )] – *p3* (3)

**Radiation in the shade**

PPFD is modulated in the case of shaded leaves:

- *p8* x PPFD_sunlit_

PPFD_shaded_ = *p7* x (1 – e ) + *r3* x PPFD_sunlit_ (4)

and total leaf area, LA, is separated into a sunlit and a shaded component.

**C assimilation**

Lastly, the amount of C produced by leaf photosynthesis during the day, C_lp_ (g day^-1^), is computed as the sum of hourly photosynthesis by sunlit and shaded leaves. k is the conversion coefficient (0.0432) from μmol CO_2_ s^-1^ to g h^-1^.

C_lp_ = [ ( P_l_^sunlit^ x LA_sunlit_ ) + ( P_l_^shaded^ x LA_shaded_ ) ] x k (5)

**Reserve Mobilisation**

If the amount of carbohydrates available from current photosynthesis is less than the amount required by the system, a mobile amount of reserves, *r4 x* CC_ls_ (g day-1), can be mobilised from the leafy shoot compartment. If it is insufficient, additional reserves from the 1-year-old stem may be used: *r5* x CC_st_. CC_ls_ and CC_st_ are the carbon content of the storage part of the leafy shoots and the 1-year-old stem, respectively. (6 and 6’)

**Maintenance respiration demand**

Maintenance respiration demand MR (g day^-1^) is calculated from the Q_10_ concept, in the same way as for the different organ groups (i), 1-year-old stem, current-year stem, leaves and fruits:

MR_i_ = *MRR*_i_ x (*Q^i^_10_*) x *DM_i_* x (3600xH) (7)

where *MRR*_i_ is the maintenance respiration rate (g g^-1^ s^-1^) of organ i at reference temperature θ_ref_ (°C), *Q^i^_10_* is the Q_10_ value for organ group i, θ is the mean temperature of the day (°C), *DM_i_* (g) is the dry mass of the organ group (i), and 3600 x H is the conversion coefficient from seconds to days. H=24 for any group except the leaves, for which only dark hours are considered.

**Fruit growth demand**

Daily carbon demand D (g day^-1^) for fruit growth can be written as:

D = x x (CC_fruit_ + *GRC_fruit_*) (8)

where Δ*DM_fruit_^pot^* / Δ*d* (g GDD^-1^) is the potential growth rate in terms of degree-days after full bloom *d*, CC_fruit_ and *GRC_fruit_* the carbon concentration and growth respiration coefficient of fruit, respectively (dimensionless). Δ*d /*Δ*t* (GDD day^-1^) is entered as a series of values in order to convert data from days to degree-days.

**Table S1.** Symbols, definitions and units of the model parameters.

|  | Parameter | **Definition** | **Unit** |
| --- | --- | --- | --- |
|  |  |  |  |
|  | *SLA* | Specific Leaf Area | m²g^-1^ |
|  | *p1* | Light-saturated maximal leaf photosynthesis | μmol CO_2_ m ^-2^ s ^-1^ |
|  | *p*  *k* | Concern leaf photosynthesis regulation by reserves | Dimensionless  Dimensionless |
|  | *p3* | Concern the calculation of leaf photosynthesis from radiation and light-saturated photosynthesis | μmol CO_2_ m ^-2^ s ^-1^ |
|  | *p4* |  | μmol CO_2_ μmol photon ^-1^ |
|  | *p7* | Concern the calculation of radiation received by shaded leaves | μmol photon m ^-2^ s ^-1^ |
|  | *p8* |  | m ² s μmol photon ^–1^ |
|  | *r3* |  | Dimensionless |
|  | *r4* | Leafy shoot mobile fraction of reserves | Dimensionless |
|  | *r5* | 1-year-old stem mobile fraction of reserves | Dimensionless |
|  | *MRR_st_^b^* | Maintenance respiration rate of 1-year-old-stem (st), current-year stem (Ost), leaf and fruit compartments at the reference temperature | gCHO g^-1^ s ^-1^ |
|  | *MRR_Ost_^b^* |  | gCHO g^-1^ s ^–1^ |
|  | *MRR_leaf_* |  | gCHO g^-1^ s ^–1^ |
|  | *MRR_fruit_* |  | gCHO g^-1^ s ^–1^ |
|  | *Q^st^_10_^b^* | Q_10_ value for 1-year-old-stem (st), current-year stem (Ost), leaf and fruit compartments | Dimensionless |
|  | *Q^Ost^_10_^b^* |  | Dimensionless |
|  | *Q^leaf^_10_* |  | Dimensionless |
|  | *Q^fruit^_10_* |  | Dimensionless |
|  | *GRC_fruit_* | Growth respiration coefficient of fruit | g g ^–1^ |

**Figure S**1. LOD profiles along the eight linkage groups of the peach genome for the 7 parameters of the model. The two horizontal lines refer to the LOD threshold calculated by permutations (black) or fixed to 1 (grey).

**Table S2**. Summary of the results of the QTL detection for the 7 parameters of the model.

| Parameters | *W_stone_* | *K_stone_* | *RGR_ini_* | *P_3_* | *A* | *B* | *SLA* |
| --- | --- | --- | --- | --- | --- | --- | --- |
| Number of QTL | 6 | 7 | 6 | 5 | 6 | 2 | 5 |
| Observed variation explained in total (%) | 50.0 | 49.6 | 21.2 | 18.4 | 16.4 | 10.0 | 19.3 |
